# GraphCrunch 2: Software tool for network modeling, alignment and clustering

**DOI:** 10.1186/1471-2105-12-24

**Published:** 2011-01-19

**Authors:** Oleksii Kuchaiev, Aleksandar Stevanović, Wayne Hayes, Nataša Pržulj

**Affiliations:** 1 Department of Computing, Imperial College, London, UK; 2 Department of Computer Science, University of California, Irvine, CA, USA

## Abstract

**Background:**

Recent advancements in experimental biotechnology have produced large amounts of protein-protein interaction (PPI) data. The topology of PPI networks is believed to have a strong link to their function. Hence, the abundance of PPI data for many organisms stimulates the development of computational techniques for the modeling, comparison, alignment, and clustering of networks. In addition, finding representative models for PPI networks will improve our understanding of the cell just as a model of gravity has helped us understand planetary motion. To decide if a model is representative, we need quantitative comparisons of model networks to real ones. However, exact network comparison is computationally intractable and therefore several heuristics have been used instead. Some of these heuristics are easily computable "network properties," such as the degree distribution, or the clustering coefficient. An important special case of network comparison is the network alignment problem. Analogous to sequence alignment, this problem asks to find the "best" mapping between regions in two networks. It is expected that network alignment might have as strong an impact on our understanding of biology as sequence alignment has had. Topology-based clustering of nodes in PPI networks is another example of an important network analysis problem that can uncover relationships between interaction patterns and phenotype.

**Results:**

We introduce the GraphCrunch 2 software tool, which addresses these problems. It is a significant extension of GraphCrunch which implements the most popular random network models and compares them with the data networks with respect to many network properties. Also, GraphCrunch 2 implements the GRAph ALigner algorithm ("GRAAL") for purely topological network alignment. GRAAL can align any pair of networks and exposes large, dense, contiguous regions of topological and functional similarities far larger than any other existing tool. Finally, GraphCruch 2 implements an algorithm for clustering nodes within a network based solely on their topological similarities. Using GraphCrunch 2, we demonstrate that eukaryotic and viral PPI networks may belong to different graph model families and show that topology-based clustering can reveal important functional similarities between proteins within yeast and human PPI networks.

**Conclusions:**

GraphCrunch 2 is a software tool that implements the latest research on biological network analysis. It parallelizes computationally intensive tasks to fully utilize the potential of modern multi-core CPUs. It is open-source and freely available for research use. It runs under the Windows and Linux platforms.

## Background

### Motivation

Many complex systems can be conveniently represented using networks. The most prominent examples are: biological, social, informational, physical and transportation networks. There are many different types of biological networks, but perhaps the most interesting of them are protein-protein interaction (PPI) networks. Proteins rarely function alone; instead they cooperate together to form complex networks of protein-protein interactions, which make our cells work. In PPI networks, nodes correspond to proteins and edges correspond to physical or functional interactions between them. The topology of PPI networks can give us new insight into the function of the individual proteins, as well as protein complexes and the whole cellular machinery as one complex system [1-7]. PPI datasets come from experimental studies such as yeast-two-hybrid (Y2H), tandem affinity purifications (TAP), high-throughput mass spectrometric protein complexes identification (HMS-PCI), and others. Recent studies have published a vast amount of PPI data for various organisms from viruses to human [8-18]. The amount of interaction data of different types and the number of organisms for which such data is available are only going to increase in the foreseeable future. Hence, the problems of biological network modeling, comparison, alignment and clustering are becoming of particular importance. To address these problems, we introduce GraphCrunch 2 - a major upgrade with added functionality to our first version of the GraphCrunch software for network analysis [19]. The following features were added to GraphCrunch 2: 1) implementations of two biologically-motivated network models: scale-free gene duplication and mutation [20] and geometric gene duplication and mutation [21] models; 2) implementation of the GRAph ALigner (GRAAL) algorithm for topological network alignment [22]; 3) implementation of a topology-based algorithm for clustering nodes in the network [6]; 4) massive computational parallelization and results reuse functionality for added computational efficiency that enables analyses of large networks data sets; and 5) an easy-to-use graphical user interface for Windows and Linux platforms.

### Previous tools for network analysis

Modeling, comparison, alignment and node clustering in complex networks are important problems across many domains and therefore several software tools addressing these problems have been introduced. In the biological network domain, some of the most commonly used ones are: Cytoscape [23], Visant [24] TopNet [25] with its successor tYNA [26], MAVisto [27], FANMOD [28], Pajek [29], Mfinder [30] with its visualization interface mDraw and the initial version of GraphCrunch [19].

Since exact network comparison is computationally intractable due to the NP-completeness of the underlying subgraph isomorphism problem, it is usually addressed by comparing various easily-computed network properties including degree distributions, clustering coefficients, and average pathlengths. tYNA [26] and Pajek [29] can be used to compute and compare these network properties, whereas Mfinder [30], MAVisto [27] and FANMOD [28] do not offer such functionality and are used for motif detection in the networks. Cytoscape, GraphCrunch [19] and now its successor GraphCrunch 2 are the only software tools that implement, in addition to basic network properties (e.g. degrees, clustering coefficients, pathlengths), highly constraining *graphlet*-based heuristics for network comparison (see Implementation section for details) [4,31]. Cytoscape does it via additional plugins that a user needs to install. In terms of network models, GraphCrunch 2 implements seven of the most commonly used random network models, whereas Cytoscape, the original version of GraphCrunch, Mfinder, FANMOD, Pajek and MAVisto implement 6, 5, 3, 3, 2 and 1 models, respectively, and other software tools do not support model generation at all.

The network alignment problem has been addressed by several local [32-35] and global [22,36-38] network alignment algorithms. Local network alignment algorithms aim to find small regions of structural similarities in two networks, whereas global network alignment algorithms align all nodes in the smaller network to nodes in the larger network. Most of these algorithms have been designed to use sequence similarity between nodes (e.g. proteins) in two networks, which makes them applicable only to networks for which such information is available, i.e., to biological networks. GRAAL, H-GRAAL, and IsoRank are the only algorithms for global network alignment that can align a pair of networks based solely on their topologies. In our previous work, we have demonstrated that GRAAL and H-GRAAL substantially outperform the IsoRank algorithm with respect to the number of aligned interactions [22,38]. Since GRAAL and H-GRAAL produce alignments of approximately the same quality [38] while GRAAL runs significantly faster, GraphCrunch 2 contains an implementation only of the GRAAL algorithm.

### Our contribution

GraphCrunch 2 is the only software that simultaneously implements methods for network modeling, comparison, alignment and topological node clustering. Moreover, it implements more random network models than any other modeling software. Also, it is the only software tool that implements all of the following: (i) pairwise network comparison using advanced *graphlet*-based heuristics; (ii) the GRAAL algorithm for network alignment, and (iii) *signature similarity*-based clustering (see Implementation section for details).

We demonstrate the utility of GraphCrunch 2 by performing two case studies. First, we use its model generation and network comparison functionality to show that eukaryotic and viral PPI networks may belong to different network model families. Second, we use its topological node clustering functionality to demonstrate a strong link between topology and function in the yeast and human PPI networks. GRAAL, its application, and comparison with other methods is found in [22].

## Implementation

### Models

Finding well-fitting network models for biological networks is an important problem in systems biology, since it can improve our understanding of biological phenomena [21], provide essential ingredients for statistical tests such as network motif identification [2], and even empower practical applications like the de-noising of PPI network [39]. The network modeling problem is formulated as follows: given a data network with *n *nodes and *m *edges, define a random graph family such that if we sample a graph with *n *nodes and *m *edges from this family (i.e. if we generate a model instance), it will be structurally similar to our data network. We discuss structural similarities between graphs in the "Network Comparison" section. GraphCrunch 2 implements the following network models: Erdős-Rényi random graphs (ER) [40], Erdős-Rényi random graphs with the same degree distribution as the data (ER-DD), scale-free Barabási-Albert preferential attachment models (SF) [1], geometric random graphs (GEO) [4,41], stickiness-index based models (STICKY) [42], scale-free gene duplication models (SF-GD) [20], and geometric gene duplication models (GEO-GD) [21].

The model network generators are implemented as follows. Erdős-Rényi random graphs are generated by using the LEDA random graph generator [43]. ER-DD graphs are generated by using the "stubs method" [44]: the number of "stubs" (to be filled by edges) is assigned to each node in the model network according to the degree distribution of the real-world network being modeled; edges are created between pairs of nodes picked at random; after an edge is created, the number of "stubs" left available at the corresponding "end-nodes" of the edge is decreased by one. Scale-free (SF) networks are generated by using the Barabási-Albert preferential attachment model [1]. Geometric random graphs are defined as follows: nodes correspond to uniformly randomly distributed points in a metric space and edges are created between pairs of nodes if the corresponding points are close enough in the metric space according to some distance norm [4,19]. In our implementation, we use boxes in Euclidean metric space, where dimensionality is a user-defined parameter. The STICKY model is based on stickiness indices, numbers that summarize node connectivities and thus also the complexities of binding domains of proteins in protein-protein interaction (PPI) networks [42]. The SF-GD and GEO-GD models are implemented by simulating gene duplication and mutation events as described in [20] and [21], respectively.

### Network Comparisons

GraphCrunch 2 uses several methods to compare the structures of two networks including: average clustering coefficients, average pathlengths, diameters, degree distributions, clustering and eccentricity spectra, [44] as well as the more constraining *graphlet*-based heuristics [4,31]. We provide the definitions of these concepts below.

GraphCrunch 2 can calculate all the following properties of a graph. The degree *deg*(*u*) of a node *u *is the number of neighbors it has in the network. The degree distribution of the network, *P*(*k*), describes the probability that a node has degree *k*. The clustering coefficient of node *u *is defined as *c*(*u*) = 2*E_u_/*(*deg*(*u*)(*deg*(*u*) - 1)), where *E_u _*is the number of edges between neighbors of *u*. For nodes with *deg*(*u*) ≤ 1, *c*(*u*) is defined to be 0 [44]. The eccentricity of node *u*, *eccen*(*u*), is the maximum shortest path distance from node *u *to some other node in the network. The average pathlength of a network is the average shortest path length across all pairs of nodes in the network. The distributions of the clustering coefficients and eccentricities of all nodes of degree *k *in a network are called the clustering and eccentricity spectra, respectively. In addition, it computes Pearson and Spearman correlation coefficients between the degree distributions, and clustering and eccentricity spectra of two networks [45]. If two distributions do not have the same length, GraphCrunch 2 disregards nodes with the smallest degrees from the larger distribution.

Recently, more constraining, *graphlet*-based heuristics have been introduced for network comparison [4,6,31]. *Graphlets *are small connected induced non-isomorphic subgraphs of a network [4]. By counting graphlets in the networks it is possible to quantify local topological similarities between networks or individual nodes. Hence, *relative Graphlet Frequency distance (RGF-distance) *is a measure that compares the frequencies of appearance of all 2 to 5 node graphlets in two networks [4]. Since there are 30 possible graphlets on up to 5 nodes, RGF-distance encompasses 30 similarity constraints by examining the fit of 30 graphlet frequencies between two networks. The smaller the RGF-distance, the more similar the two networks are [4]. From a topological point of view, it is relevant to distinguish between *automorphism orbits *of each graphlet. For example, in a 3-node path, the "end-nodes" are identical from the topological point of view (i.e., can be mapped to each other by an *automorphism*, an *isomorphism *of a graph with itself-see [31] for details), whereas the "middle node" is different; therefore, a 3-node path has two different automorphism orbits. There are 73 automorphism orbits for the 30 graphlets on 2 to 5 nodes. *The Graphlet degree vector *(or *signature*) of node *v *is a 73-component vector, such that its *i^th ^*coordinate represents number of times this node is touched by an automorphism orbit *i*. The signature of a node is a highly constraining measure of local topology in the node's vicinity and comparing the signatures of two nodes is a highly constraining measure of local topological similarity between them [6,31]. *The Graphlet Degree Distribution agreement (GDD-agreement) *is a similarity measure between topologies of two networks based on graphlet degree vector distributions (see [31] for details). That is, it is used to compare the structural similarities between two networks. It is a number between 0 and 1 meaning that two networks are similar if they have high GDD-agreement.

### Network Alignment

Sequence comparison and alignment has had an enormous impact on our understanding of evolution, biology and disease. Comparison and alignment of biological networks will probably have a similar impact and hence network alignment is a foremost problem in systems biology [46]. Existing network alignments use information external to the networks, such as sequence. Our algorithm, GRAAL [22], uses purely topological information based on graphlets in order to perform network alignment. Since we use only topological information, GRAAL can be applied to *any *two networks, not just biological ones. We have applied GRAAL to biological networks to produce by far the most complete topological alignments of biological networks to date [22]. In that paper we demonstrated that both species phylogeny (i.e. phylogenetic trees similar to ones produced by sequence) and detailed biological function of individual proteins can be extracted from our alignments. Topology-based alignments can provide a completely new, independent source of phylogenetic information. Furthermore, we aligned the protein-protein interaction networks of two very different species-yeast and human-and found that even these two distant species share a surprising amount of network topology, and that aligned protein pairs share a significant amount of biological similarity. This provides strong evidence for broad similarities in internal cellular wiring across all life on Earth.

It has been shown that pairwise network alignment algorithms, including our algorithm called GRAAL that is implemented in GraphCrunch 2 [22], can be used to successfully address the following important problems:

1. Finding conserved modules in PPI networks of different species [22,36,38,46]

2. Identifying functional orthologs in PPI networks of different species [22,36,38]

3. Reconstructing phylogenetic relationships among a group of species [22,38]

For these reasons, we have added an easy-to-use interface to GRAAL [22] in GraphCrunch 2. Given two networks, GRAAL finds an "embedding" of the smaller network into the larger one such that every node in the smaller network is aligned to exactly one node in the larger one. The goal is to expose as much topological similarity between the networks as is possible. GRAAL is a seed-and-extend algorithm that greedily aligns nodes based on their signature similarities while traversing both networks simultaneously in a breadth- first manner. In [22] it was shown that GRAAL produces *topological *alignments that expose regions of *functional *similarity that are far larger, denser, and superior in many ways to other available methods. Note however, that GRAAL should not be used for aligning noisy biological networks, since it uses topology only and hence it is not be expected to align such data correctly. Instead, it should be used to align as clean and complete biological networks as possible, e.g. high-confidence parts of PPI networks, metabolic networks of closely related species, or protein structure networks.

Since GRAAL is based solely on network topology, it is applicable to all types of networks. However, such generality comes with a price GRAAL does not utilize any additional information which might be available about nodes in the networks (e.g., sequence similarity, structural similarity, etc.). Even though it may seem easy to add such information to the GRAAL algorithm [22], finding an elegant and scientifically sound way of doing it is a subject of future research.

### Clustering

It is has been shown that similar interaction patterns imply functional similarities between proteins [6,47,48]. Hence, one way to detect functionally similar proteins in a network is to cluster its nodes based on their topological similarities. Also, such clustering might provide insights into how proteins perform their functions by interacting with each other. GraphCrunch 2 contains an easy-to-use implementation of the *k*-medoids algorithm for clustering nodes in the network with a signature-based distance matrix [47]. The algorithm works as follows. It randomly initializes *k *cluster centers and then assigns the remaining nodes in the network to the clusters represented by nearest centers. Then cluster centers are recomputed and the process is repeated until convergence. The only difference between *k*-medoids and the more conventional *k*-means algorithm [45] is that the latter uses means of points in the cluster to represent the new cluster center, while the former requires all cluster centers to be the *existing data points *(nodes from the network). This minor difference has an important implication: in the case of *k*-medoids, a network's nodes do not have to be represented as points in some metric space. All we need to have in order to apply the *k*-medoids algorithm is an all-to-all matrix of distances between nodes. GraphCrunch 2 can load any user-provided distance matrix, or it can automatically compute all-to-all *signature similarities *between nodes in the network and use as the distance between any two nodes *u *and *v*, *D*(*u, v*) = 1 - *S*(*u, v*), where *S*(*u, v*) is the signature similarity between *u *and *v*.

Among signature similarity-based methods that produce non-overlapping clusters (hierarchical clustering, *k*-medoids, and signature threshold-based clustering), *k*-medoids has been shown to produce the best results [47]. Therefore, we chose this algorithm as our default implementation. In addition, in our "Case study 2" section below we use the human PPI network to demonstrate that our clustering method outperforms the Markov Cluster Algorithm (MCL) [49,50].

Finally, GraphCrunch 2 allows several clustering scenarios. If the user wishes to use a signature similarities matrix with any other clustering algorithm, GraphCrunch 2 can compute and save such a matrix into a file for future usage with other clustering software. In addition, such similarity matrix can be constructed for a pair of different networks, if the user wants to analyze topological similarities across different networks.

### Development

We developed GraphCrunch 2 in C++ using the QT framework 4.6 to allow the same user experience and high performance across popular Windows and Linux distributions [51]. Additionally, GraphCrunch 2 uses the free edition of LEDA 6.2 [43] and Qwt 5.2 libraries [52]. The LEDA library is used for standardized handling of network input and basic computational analysis, while the Qwt library provides plotting capabilities.

We tested GraphCrunch 2 under Microsoft Windows (XP, Vista, Windows 7) and Linux (Ubuntu 9.10 and Arch Linux) operating systems. GraphCrunch 2 can be compiled (and we provide the binaries on the website) for 32- and 64-bit platforms.

#### User Interface

We designed GraphCrunch 2 with two main goals in mind: 1) to integrate and simplify the most important tasks of biological network analyses and 2) to provide an intuitive and easy to use graphical user interface for people whose primary area of expertise is not computer science, enabling them to run computationally expensive analyses on their laptops.

**Figure 1:**
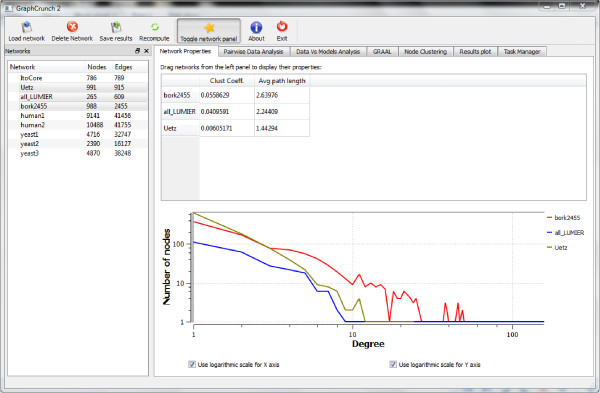
**GraphCrunch 2 user-interface**. GraphCrunch 2 user-interface screenshot. Using the main menu, the user can load and delete networks, and save the results of the analysis. The "Networks" panel on the left contains a list of networks currently loaded into GraphCrunch 2. The right hand side of the user interface is a tab-based control that contains several tabs corresponding to different features implemented in GraphCrunch 2. The "Network Properties" tab is used to compute the basic properties of the data, such as the average pathlength and the average clustering coefficient, and display the degree distribution of the data. The "Pairwise Data Analysis" tab is used to perform pairwise comparison of the data networks. The "GRAAL" tab is used to align any two networks using the GRAAL algorithm. The "Node Clustering" tab is used to compute all-to-all signature similarities between nodes in any pair of networks and to topologically cluster nodes in a network. the "Results plot" tab is used for plotting the results. the "Task Manager" tab displays the list of all generated basic tasks and their current status.

To achieve the second goal we created a simple, user-friendly graphical user interface (GUI) depicted in Figure 1. Also, we have taken full advantage of the intuitive drag-and-drop functionality to simplify many common tasks. Finally, our implementation follows the Model/View/Controller architecture to separate the GUI from the application logic and, therefore, make GraphCrunch 2 easily expandable.

#### Input and output

GraphCrunch 2 supports two input formats for representing networks: the LEDA graph format (.gw) [43] and the "edge list" format (.txt and .edgeLst). The edge list format is simply the graph adjacency list, i.e., the list of node pairs (edges of the network) separated by tabs or spaces, with one edge per line. GraphCrunch 2 automatically converts from edge list to LEDA graph format. The current implementation of GraphCrunch 2 deals with undirected, simple (i.e., no loops or multiple edges), unweighted graphs. Thus, for either of the above two formats, GraphCrunch 2 automatically removes all self-loops, multiple edges and edge directions.

The summarized output statistics of each analysis completed by GraphCrunch 2 can be saved in either of the following two formats: comma-separated format (.csv) or tab-separated format (.tsv). Additionally, the user can copy/paste the resulting tables into a separate application for further analysis. If the user clicks on the "Save results" button in the Main Menu, GraphCrunch 2 will save the results from the currently active tab. Also, GraphCrunch 2 has a basic plotting functionality, being able to plot the degree distributions of the data networks, GDD-agreement, RGF-distance and other statistics of the data versus models or pairwise data analyses. Furthermore, the plotting functionality allows the user to select subsets of the results to be plotted, allowing the user to focus on results that are of interest. Our plots can be exported in .png format, or as a table; the latter can be used by other programs for producing plots in other formats, if desired.

#### Parallelization

GraphCrunch 2 automatically parallelizes many complex tasks by splitting them into smaller independent tasks that can be run in parallel. GraphCrunch 2 automatically determines the number of logical processors/cores available and runs as many basic tasks as is practical. More specifically, complex tasks (i.e. finding the best fitting model for the data, network comparison and alignment, clustering) are split into the following 8 basic tasks: model generation, counting graphlets, computing RGF-distance, computing GDD-agreement, GRAAL execution, computing signature similarities, *k*-medoids clustering and comparing basic network properties. It is important to note that there might be multiple dependencies between tasks. For example, before computing GDD-agreement between two networks, GraphCrunch 2 must finish counting graphlets in both networks. Since all such dependencies can be predetermined at runtime, GraphCrunch 2 automatically calculates dependencies between tasks and schedules them for parallel execution without any intervention from the user.

For example, if the user has 3 data networks and wants to determine which model best fits the data by comparing the data networks to 10 instances of each model, GraphCrunch 2 will split the computations into the following basic tasks: model generation (1 task for each data network; for each model type, 7 models are supported, and for each model network instance; hence 3 × 7 × 10 = 210 tasks), counting graphlets (separate tasks for each data network and generated model network 3 + 3 × 7 × 10 = 213 tasks), computing GDD-agreement, RGF-distance and basic properties for each pair of data-model networks (3 × 3 × 7 × 10 = 630 tasks). Hence, in total such analysis is split into 1,053 basic tasks; however at any given moment in time, GraphCrunch 2 runs only as many tasks as there are CPU cores available given that all their dependencies are satisfied.

GraphCrunch automatically saves the results of each basic task for possible re-use in subsequent analyses (if applicable). The user can interrupt and then resume any analyses from practically any point without losing or recomputing any results.

### GraphCrunch 2 performance

The following tests use a ThinkPad with an Intel Core i3 CPU (4 cores), 4Gb of RAM and Windows 7.

1. Comparing 5 viral PPI networks [18] against all models (30 instances per model): about 4 hours.

2. Aligning the yeast (16, 127 interactions amongst 2,390 proteins) [16] and human (41,456 interactions amongst 9, 141 proteins) [53] PPI networks using GRAAL: about 3 hours. (See [22] for a discussion of the biological significance of this alignment.)

3. Aligning any pair of the viral PPI networks listed in [18] takes several seconds. Performing all-to-all pairwise data comparison (i.e. computing and comparing all the parameters for all pairs of networks) of these networks also takes several seconds.

4. Clustering nodes in the high-confidence yeast PPI network (9, 074 interactions amongst 1,622 proteins) [16] takes about 2 hours with the most of the time being spent on counting graphlets. Once counting of the graphlets is done, GraphCrunch 2 can re-use these results and cluster the nodes in this network in about 2 minutes for any *k *which we analyzed (we varied *k *from 5 to 50).

As mentioned above, the most computationally intensive basic task in GraphCrunch 2 is counting of graphlets [4,19]. The time complexity of counting of graphlets is *O*(|*V*|^5^). However, as it has been shown before [19,54], the running time of graphlets count depends both on the size of the network and on its density, with sparser networks being processed much faster than the denser ones. Since PPI networks are known to be sparse, and since GraphCrunch 2 has parallel computing capabilities, it has competitive performance for processing the currently available PPI networks.

## Results and Discussion

### Case study 1: Modeling viral PPI networks

**Figure 2:**
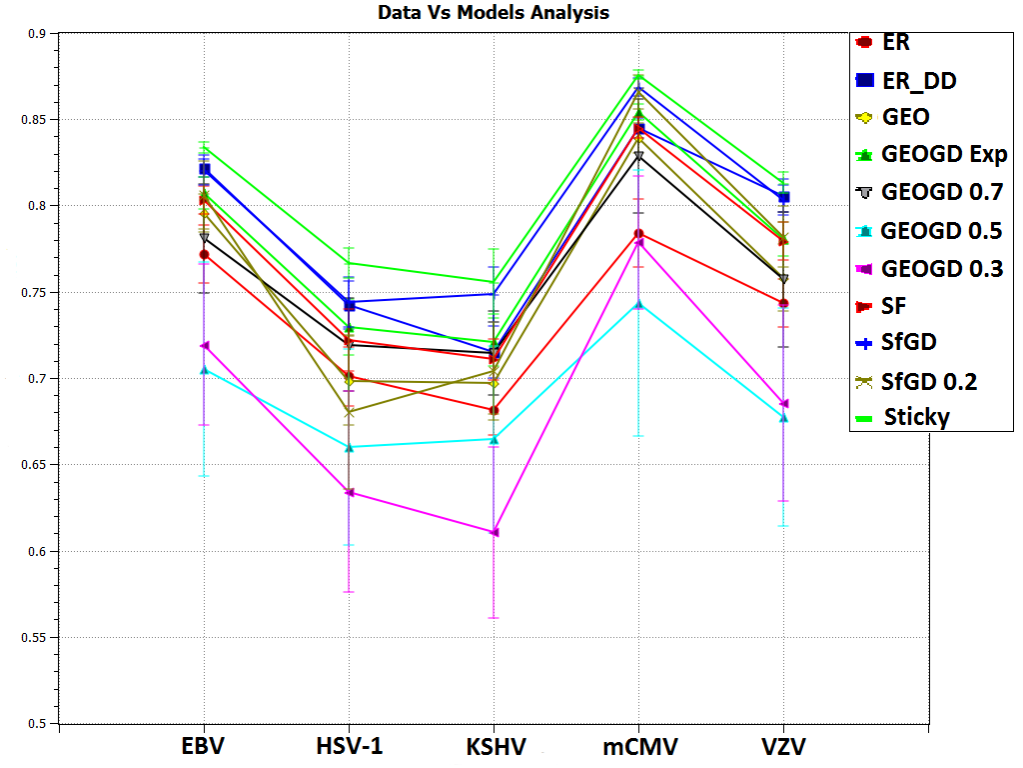
**GDD-agreement between the viral PPI networks and the model networks**. GDD-agreement between the viral PPI networks and the model networks. Points on the horizontal axis correspond to viral PPI networks of: EBV, HSV-1, KSHV, mCMV, and VZV herpes viruses. Lines with different labels correspond to different model networks. The vertical axis represents the average GDD-agreement between the corresponding data and model networks. For each data network, we generated 30 network model instances, each constrained to be of the size of the data. The plot reports the averaged the GDD-agreement values and standard deviations of their GDD-agreement with the data. Higher values of GDD-agreement reflect higher similarities between the networks. This plot was generated by GraphCrunch 2.

**Figure 3:**
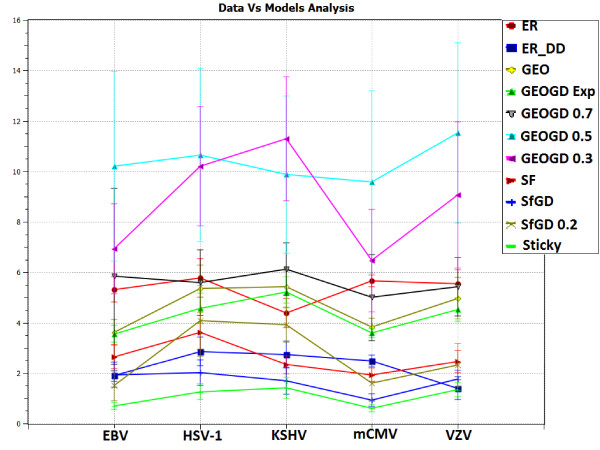
**RGF-distance between viral PPI and model networks**. RGF-distance between the viral PPI networks and the model networks. Points on the horizontal axis correspond to viral PPI networks of: EBV, HSV-1, KSHV, mCMV, and VZV herpes viruses. Lines with different labels correspond to different model networks. The vertical axis represents the average RGF-distance between the corresponding data and model networks. Thirty instances of each network model were generated for each data network and constrained to be of the size of the data and averaged RGF-distance values and standard deviations of their RGF-distance with the data are reported on the plot. Higher values of RGF-distance reflect lower similarities between the networks. This plot was generated by GraphCrunch 2.

We analyze viral PPI networks of: varicella-zoster virus (VZV), Kaposi's sarcoma-associated herpesvirus (KSHV), herpes simplex virus 1 (HSV-1), Epstein-Barr virus (EBV) and murine cytomegalovirus (mCMV) [18]. Fossum *et al*. noticed that PPI networks of these viruses differ from PPI networks of eukaryotic organisms (including yeast, fruit fly, worm and human). Among the reported differences were: different degree distributions, smaller clustering coefficients and higher attack tolerance and robustness in the sense of smaller loss of connectivity in response to random node deletions [18]. Hence, it is natural to ask if viral and eukaryotic PPI networks belong to the same network model. We use GraphCrunch 2 to demonstrate that these viral PPI networks may belong to a different network family than do PPI networks of the above mentioned eukaryotic organisms. This is the first time that this has been demonstrated. Using GraphCrunch 2, we compare the five viral PPI networks with 30 random instances of each of the following network models that are of the size of the data networks: ER [40], ER-DD, SF [1], GEO [4,41], STICKY [42], SF-GD [20], and GEO-GD [21] (see Models section for details). Figure 2 and 3 present GDD-agreement and RGF-distance between the viral PPI networks and the corresponding model networks. For clarity, in these figures we present GEO-GD probability cutoff model only for *p *= 0.5, 0.3 and 0.7 and SF-GD model only for *q *= 0.5 and 0.2. As it follows from Figure 2 and 3, according to both GDD-agreement and RGF-distance, the best-fitting model for viral PPI networks is the stickiness-index based model (STICKY) [42].

This result is interesting because we have previously demonstrated that the best-fitting model (with respect to GDD-agreement and RGF-distance) for high-quality PPI networks of eukaryotic organisms is the geometric gene duplication model (GEO-GD) [21]. Hence, the differences in simple network properties between eukaryotic and viral interactomes noticed by Fossum *et al. *[18] may be attributable to their belonging to different network models.

### Case study 2: Topological clustering of nodes in the human PPI network

Many studies have shown that there is a link between PPI network topology and biological function [2,6,22,36,38,50]. It has been demonstrated that signature similarity-based clustering produces biologically meaningful clusters [6,47]. In particular, it can be used for protein function prediction [6] and cancer gene identification [47].

The intuition behind signature similarity-based clustering is as follows. In order to perform their functions, proteins interact with each other and, therefore, topologically similar interaction patterns should result in functional similarities between proteins. Since signature similarity is a very powerful measure of topological similarity between nodes in a network [6], we use signature-based clustering implemented in GraphCrunch 2 to check if topological similarities in nodes' neighborhoods indeed result in biological similarities.

We take the human PPI network published by Radivojac *et al. *[53] (which consists of 41, 456 interactions amongst 9,141 proteins) and cluster its nodes using GraphCrunch 2 into *k *= 10, 20, 30, 40, 50, 60, 70, 80, 90, and 100 clusters (the clusters are available from the GraphCrunch 2 website). We use the Gene Ontology (GO) database to see if our clusters contain functionally similar proteins [55]. We perform our analyses considering only "biological process" GO terms. Hence, we exclude from our analysis GO terms corresponding to "molecular function" and "cellular component." We do so because "cellular component" GO terms might be too general in this context and "molecular function" terms reflect proteins' chemical properties rather than their biological functions. Hence, for each *k*, we analyze GO term enrichment of each cluster [55].

To analyze the statistical significance of GO term enrichments of our clusters we use the GO Terms Finder (version 0.86) software package [56]. This software tool examines a list of proteins to determine if there is a set of GO terms, or parents of those GO terms (since the GO ontology is organized as a tree), that is shared by a statistically significant fraction of proteins in the list. Hence, for each *k *we run GO Term Finder on each of the *k *clusters to determine which GO terms are shared by a statistically significant fraction of proteins in the cluster. The GO Terms Finder calculates p-values based on the model of sampling without replacements using the hypergeometric distribution. Therefore, in order to obtain valid p-values, we use as the background set not the set of all yeast proteins, but only those proteins which are present in the human PPI network that we analyze [53]. Also, GO Terms Finder performs Bonferroni adjustment for its p-values and below we report only Bonferroni-adjusted p-values; for details see [56]. We downloaded the GO annotation data for this case study on May 1, 2010 from the Gene Ontology website [55]. In our experiments, we used 0.01 as the statistical significance cut-off for our p-values.

The results of this analysis are presented in Table 1. Below we elaborate on several examples from Table 1. For *k *= 10, there are 9 clusters that are statistically significantly enriched with GO terms. In total, 693 "biological process" GO terms are shared by a statistically significant fraction of proteins in at least one of these clusters. For example, there is a cluster in which 158 out of 283 proteins are responsible for positive regulation of cellular process (GO:0048522), with the p-value of 5 × 10^-31^. In the same cluster, 149 proteins are in a signaling pathway (GO:0023033), with the p-value of 2.5 × 10^-30^. In total, this cluster is statistically significantly enriched with 377 GO terms (some of which are "parent" terms of the others). In another cluster, 16 out of 183 proteins are responsible for negative regulation of ubiquitin-protein ligase activity during mitotic cell cycle (GO:0051436), with the p-value of 1.7 × 10^-10 ^because only 61 proteins are annotated with this GO terms in the PPI network. In total, this cluster is enriched with 39 different GO terms.

**Table 1:** Clustering nodes in the human PPI network

*k*	# Significant Clusters	# Significant Terms	Best Enrichment	# Best Clusters
10	9	693	92.58%	1
20	14	694	95.16%	1
30	17	777	97.87%	1
40	24	814	94.08%	1
50	22	855	94.08%	1
60	24	860	97.01%	1
70	28	852	93.92%	1
80	40	892	94.11%	1
90	38	862	100%	1
100	44	867	98%	1
115	49	873	98%	1

*k*-medoids clusters statistics for the human PPI network [53]. The first column corresponds to the number of clusters (*k*) used in the *k*-medoids algorithm. The column labeled "# Significant Clusters" contains the number of clusters for which at least one "biological process" GO term is shared by a statistically significant fraction of proteins in that cluster. The column "# Significant Terms" contains the number of "biological process" GO terms that are shared by a statistically significant fraction of proteins in at least one cluster. The column "Best Enrichment" contains the highest percentage of proteins in some cluster that have the same "biological process" GO term. The column "# Best Clusters" contains the number of clusters corresponding to the best enrichment.

For *k *= 100, there are 44 clusters statistically significantly enriched with GO terms. For example, there is cluster of 46 proteins that contains 5 proteins responsible for blood coagulation (GO:0030193) and wound healing, with the p-value of 8.5 × 10^-4^. Another cluster of 34 proteins contains 5 (out of 11 in the network) proteins responsible for drug metabolic process (GO:0017144), with the p-value of 7.9 × 10^-8^; 3 of these 5 proteins (out of 5 in the network) are also responsible for exogenous drug catabolic process (GO:0042738), with the p-value of 1.2 × 10^-4^. In another cluster of 35 proteins there are 6 proteins (out of 33 in the network) that are responsible for tRNA processing (GO:0008033), with the p-value of 8.4 × 10^-7^. There is a cluster in which 8 out of 32 proteins are responsible for negative (GO:0051436) and positive (GO:0051437) regulation of ubiquitin-protein ligase activity during mitotic cell cycle with p-values lower than 2.1 × 10^-8^. In general, there are 867 GO terms which are shared by a statistically significant fraction of proteins in at least one of these clusters.

To further explore the biological significance of our clusters, for each *k*, we examine each cluster in the human network with respect to the enrichment with proteins corresponding to: aging genes http://genomics.senescence.info/, HIV-1 interacting genes http://www.ncbi.nlm.nih.gov/RefSeq/HIVInteractions/, pathogen-interacting genes http://staff.vbi.vt.edu/dyermd/publications/dyer2008a.html, and cancer-related genes [6]. To determine the significance of enrichments, we compute p-values using the hyper-geometric distribution and use the p-value cutoff of 0.01. For *k *= 100, there are 4 clusters statistically significantly enriched with aging genes, 13 clusters statistically significantly enriched with HIV-1 interacting genes, 10 clusters statistically significantly enriched with pathogen-interacting genes, and 8 clusters statistically significantly enriched with cancer-related genes. Interestingly, there is one particular cluster of 183 proteins that is statistically significantly enriched with genes from all 4 categories. In particular, 39.34% of the proteins in that cluster correspond to aging genes, 63.93% of the proteins correspond to HIV-1 interacting genes, 33.88% of the proteins correspond to pathogen interacting genes, and 36.06% of the proteins correspond to cancer related genes (the maximum p-value is 1.34 × 10^-11^). According to GO Terms Finder, this particular cluster is statistically significantly enriched with 366 different GO terms (some of them are parents of others), among which are: interspecies interaction between organisms (GO:0044419, p-value 2.43 × 10^-18^), regulation of signaling pathway (GO:0035466, p-value 2.14 10^-18^), regulation of cell death (GO:GO:0010941, p-value 1.42 × 10^-22^) and 9 other death-related terms (GO:0043067, GO:0060548, GO:0043069, GO:0043068, GO:0010942, GO:0012502, GO:0012501, GO:0008219, GO:0016265).

To confirm that our clustering does in fact capture a meaningful biological signal, we perform random clustering of the same human PPI networks into the same numbers of clusters as described above. Then, we analyzed these random clusters with Go Term Finder software [56]. Not surprisingly, there is no biological signal in these random clusters. For *k *= 50, 60, 70, and 90 GO Term Finder reported statistically significant enrichment with GO terms for 2, 1, 3, and 1 clusters, respectively, with the maximum enrichment with 4 GO terms (compared to the minimum enrichment with 693 terms, when we used GraphCrunch 2). Given the amount of generated clusters (550) and the amount of GO terms in the database, this can be expected at random. Therefore, the amount of "false" biological signal in our clusters is minimal.

As with many other clustering algorithms, the *k*-medoids algorithm implemented in GraphCrunch 2 requires the number of clusters to be predefined in advance by the user. Determining the right number of clusters is an important research question, and its value depends on the particular situation. We suggest trying several *k*'s and examining all configurations for biological meaningfulness using, for example, GO Term Finder (as we did above) [56].

The Markov Cluster Algorithm (MCL) is another popular graph clustering algorithm which can cluster graphs based solely on their topology [49,50]. Unlike *k*-medoids, it does not require the number of clusters to be given in advance; however, it requires an *inflation *(or granularity) parameter [49]. We ran MCL (the latest version (20 Jul 2010 mcl 10-201) from http://micans.org/mcl/) on the same human PPI network using the inflation parameter *I *from 1.2 to 4, as recommended. For *I *= 1.2, the algorithm produced 115 clusters while for other values of the parameter (≥ 1.5) it produced more than 2,700 clusters (for clustering 9,141 nodes in the network) many of which contained only 1 or 2 nodes. We used GraphCrunch 2 to produce 115 clusters and compared their GO term enrichment with that of the clusters produced with MCL (for *I *= 1.2), using GO Terms Finder. Out of 115 clusters produced by GraphCrunch 2, 49 were found to be statistically significantly enriched with 875 GO terms. Also, the smallest cluster produced by GraphCrunch 2 had 6 proteins. In the clustering produced by MCL, 76 out of 115 contained 2 proteins and therefore it is not possible to judge the statistical significance of their GO term enrichment. Out of the remaining 39 clusters, GO Terms Finder found 25 clusters to be statistically significantly enriched with 333 GO terms. Hence, for this particular network, clustering produced by GraphCrunch 2 has twice as many significantly enriched clusters and with more GO terms. Thus, GraphCrunch 2 appears to produce better results than MCL for these particular datasets. In addition, it produces better results than hierarchical or signature threshold-based clustering [47].

We also compare the performance of our method with that of Aragues et al. [57], which also predicts from PPI networks the involvement of genes in cancer. While [57] focus only on direct network neighbours of cancer genes, we account for complex wirings of their up to 4-deep neighbourhoods, using 5-node graphlets; we demonstrate [47] that out of all known cancer gene pairs that have similar topological signatures, 96 percent are not direct neighbours in the PPI network. Moreover, in addition to network topology, [57] also use gene expression data and structural and functional properties of cancer proteins, while we use the network topology only. Even though we do not use any information external to PPI network topology, our approach is superior, as it results in higher prediction accuracy [47]. Thus, graphlet degree signatures provide a better prediction accuracy than less constraining network properties such as nodes' direct neighbours, even when nodes' direct neighbourhoods are integrated with other data types.

### Comparison with existing tools

We compare GraphCrunch 2 with eleven of the most commonly used tools: the initial version of GraphCrunch [19], Cytoscape [23], Visant [24] Mfinder [30], MAVisto [27], FANMOD [28], tYNA [26], Pajek [29], IsoRank [36], Graemlin [34], and GraphM [37]. The summary of the functionality of these tools is presented in Table 2. Cytoscape is the closest in functionality to GraphCrunch 2, but GraphCrunch 2 has the following unique features. None of the tools other than GraphCrunch 2 can compute signature similarities between nodes in a network, or between two networks. Cytoscape has a plugin "GraphletCounter" that can compute node signatures, but it cannot find signature similarities between nodes, cluster nodes based on these similarities, or compare networks based on RGF-distances or GDD-agreements (thus, we put "Limited" in the entry of Table 2). Hence, GraphCrunch 2 is the only software tool that can cluster network nodes based on their topological signature similarities. Furthermore, GraphCrunch 2 is the only tool that can compare real networks to one another or to model networks based on RGF-distances and GDD-agreements. Note that mFinder finds network motifs, not graphlets. GraphCrunch 2 is the only software tool that implements the GRAph ALigner (GRAAL) algorithm; this is in addition to its ability to generate model networks, compute graph properties (global and local) and comparing networks based on them, visualizing the results, computing node signature similarities and performing topological node clustering. Also, GraphCrunch 2 offers the largest number of network models. With regards to clustering, GraphCrunch 2 implements *k*-medoids applied to graphlet-based node signature similarity (see Clustering section above); this clustering method has been shown to outperform hierarchical clustering, signature threshold-based clustering [47] and the MCL algorithm (see Case study 2 above). Cytoscape has plugins for many clustering methods, but it cannot cluster nodes based on their topological signatures. Also note that Cytoscape and its plug-ins are all written in Java, while GraphCrunch 2 is written in compiled C++. For example, computing all signatures for a network with 1,278 nodes and 1,809 edges in Cytoscape takes about 5 minutes, while GraphCrunch 2 can do this and cluster this network in about 1 minute. Furthermore, compared to GraphCrunch 2, Cytoscape lacks task-based parallelization features for counting graphlets (e.g. simultaneously count graphlets in more than 1 network). Hence, it is not able to provide fast processing of larger data sets by compute intensive algorithms, such as graphlet and orbit counts, or network alignment. We further note that Cytoscape is a plugin-based platform and therefore, to enable certain features, the user needs to install additional plugins; to summarize its functionality in comparison with other tools in Table 2 we used a list of all available plugins from http://chianti.ucsd.edu/cyto_web/plugins/.

**Table 2:** Comparison of software tools for biological network analysis

Software package	Graph Properties	#of models	Graphlets	Visualization	Clustering	GNA
GraphCrunch 2	Yes	7	Yes	Yes (Results)	Yes	Yes
GraphCrunch	Yes	5	Yes	Yes (Results)	No	No
Cytoscape	Yes	6	Limited	Yes	Yes	No
Visant	Yes	1	No	Yes	No	No
mFinder	No	3	No	Yes (mDraw)	No	No
MAVisto	No	1	No	Yes	No	No
FANMOD	No	3	No	Yes	No	No
tYNA	Yes	0	No	Yes	No	No
pajek	Yes	2	No	Yes	Yes	No
IsoRank	No	0	No	No	No	Yes
Graemlin	No	0	No	No	No	Yes
GraphM	No	0	No	No	No	Yes

The "Graph Properties" column contains "Yes" if the package can compute at least the average diameter and clustering coefficient. The "# models" column corresponds to the number of random network model families supported by the package. The "Graphlets" column contains "Yes" if the package can count all graphlets and orbits in the network with up to 5 nodes and if it can compute signature similarities. The "Visualization" column contains "Yes" if the package is able to visualize graphs (graph drawing) or results (statistics). The "Clustering" and "GNA" Columns indicate whether the package supports clustering and Global Network Alignment (GNA), respectively.

## Conclusions

GraphCrunch 2 is a software tool that implements the latest research on biological network analyses. GraphCrunch 2 includes implementations of network modeling, comparison, alignment and clustering. We have demonstrated that GraphCrunch 2 can be used to extract biological information from the network topology. We believe that our case studies barely scratch the surface of the knowledge that can be extracted from the interaction data. As more interaction data for multiple species are becoming available, software tools such as GraphCrunch 2 will become increasingly useful.

## Availability and requirements

**Project name**: GraphCrunch 2

**License**: GNU GPL

**Project homepage**: http://bio-nets.doc.ic.ac.uk/graphcrunch2/

**Operating systems **Microsoft Windows (XP, Vista, 7), Linux

**Programming language**: C++

## Authors' contributions

OK and AS contributed to the design and implementation of GraphCrunch 2 functionality. OK performed all statistical test. WH designed and implemented the initial version of the GRAAL alignment algorithm. NP directed all aspects of the research. All authors read and approved the manuscript.
